# A multidisciplinary program for meningioma treatment, research, and support services

**DOI:** 10.1093/nop/npag051

**Published:** 2026-05-26

**Authors:** Zora Arum, Ramin A Morshed, Brooke C Braman, Kanish Mirchia, Javier E Villanueva-Meyer, Vaughn Mouton, Tracy Ward, Charlotte Huie, Jennifer Viner, Alon Witztum, Molly Rose Morrissey, Ashley Bazell, Stephanie Lewis, Linda Pham, Sasha Mortezaei, Melike Pekmezci, Jacob S Young, Lauren Boreta, Matthew S Susko, Jean L Nakamura, Steve E Braunstein, Marc H Levin, Shawn L Hervey-Jumper, Tarun Arora, Manish K Aghi, Tina R Jimenez, Christina Weyer-Jamora, Arie Perry, Penny K Sneed, John F de Groot, Edward F Chang, Naomi Hoffer, Susan M Chang, Mitchel S Berger, Michael W Mcdermott, Philip V Theodosopoulos, William C Chen, Nancy Ann Oberheim Bush, David R Raleigh

**Affiliations:** Department of Neurological Surgery, University of California San Francisco, San Francisco, CA, USA; Department of Pathology, University of California San Francisco, San Francisco, CA, USA; Department of Radiation Oncology, University of California San Francisco, San Francisco, CA, USA; Department of Neurological Surgery, University of California San Francisco, San Francisco, CA, USA; Department of Neurological Surgery, University of California San Francisco, San Francisco, CA, USA; Department of Pathology, University of California San Francisco, San Francisco, CA, USA; Department of Radiation Oncology, University of California San Francisco, San Francisco, CA, USA; Department of Pathology, University of California San Francisco, San Francisco, CA, USA; Department of Neurological Surgery, University of California San Francisco, San Francisco, CA, USA; Department of Radiology, University of California San Francisco, San Francisco, CA, USA; Department of Neurological Surgery, University of California San Francisco, San Francisco, CA, USA; Department of Neurology, University of California San Francisco, San Francisco, CA, USA; Division of Neuro-Oncology, University of California San Francisco, San Francisco, CA, USA; Department of Neurological Surgery, University of California San Francisco, San Francisco, CA, USA; Department of Neurological Surgery, University of California San Francisco, San Francisco, CA, USA; Department of Neurological Surgery, University of California San Francisco, San Francisco, CA, USA; Department of Radiation Oncology, University of California San Francisco, San Francisco, CA, USA; Department of Neurological Surgery, University of California San Francisco, San Francisco, CA, USA; Department of Neurological Surgery, University of California San Francisco, San Francisco, CA, USA; Department of Neurology, University of California San Francisco, San Francisco, CA, USA; Division of Neuro-Oncology, University of California San Francisco, San Francisco, CA, USA; Department of Neurological Surgery, University of California San Francisco, San Francisco, CA, USA; Department of Neurological Surgery, University of California San Francisco, San Francisco, CA, USA; Department of Pathology, University of California San Francisco, San Francisco, CA, USA; Department of Neurological Surgery, University of California San Francisco, San Francisco, CA, USA; Department of Radiation Oncology, University of California San Francisco, San Francisco, CA, USA; Department of Radiation Oncology, University of California San Francisco, San Francisco, CA, USA; Department of Radiation Oncology, University of California San Francisco, San Francisco, CA, USA; Department of Radiation Oncology, University of California San Francisco, San Francisco, CA, USA; Department of Ophthalmology, University of California San Francisco, San Francisco, CA, USA; Department of Neurological Surgery, University of California San Francisco, San Francisco, CA, USA; Department of Neurological Surgery, University of California San Francisco, San Francisco, CA, USA; Department of Neurological Surgery, University of California San Francisco, San Francisco, CA, USA; Division of Neuro-Psychology, University of California San Francisco, San Francisco, CA, USA; Division of Neuro-Psychology, University of California San Francisco, San Francisco, CA, USA; Department of Pathology, University of California San Francisco, San Francisco, CA, USA; Department of Radiation Oncology, University of California San Francisco, San Francisco, CA, USA; Department of Neurological Surgery, University of California San Francisco, San Francisco, CA, USA; Department of Neurology, University of California San Francisco, San Francisco, CA, USA; Division of Neuro-Oncology, University of California San Francisco, San Francisco, CA, USA; Department of Neurological Surgery, University of California San Francisco, San Francisco, CA, USA; Department of Neurological Surgery, University of California San Francisco, San Francisco, CA, USA; Division of Neuro-Oncology, University of California San Francisco, San Francisco, CA, USA; Department of Neurological Surgery, University of California San Francisco, San Francisco, CA, USA; Division of Neuro-Oncology, University of California San Francisco, San Francisco, CA, USA; Department of Neurological Surgery, University of California San Francisco, San Francisco, CA, USA; Department of Neurological Surgery, University of California San Francisco, San Francisco, CA, USA; Department of Neurological Surgery, University of California San Francisco, San Francisco, CA, USA; Department of Radiation Oncology, University of California San Francisco, San Francisco, CA, USA; Department of Neurological Surgery, University of California San Francisco, San Francisco, CA, USA; Department of Neurology, University of California San Francisco, San Francisco, CA, USA; Division of Neuro-Oncology, University of California San Francisco, San Francisco, CA, USA; Department of Neurological Surgery, University of California San Francisco, San Francisco, CA, USA; Department of Pathology, University of California San Francisco, San Francisco, CA, USA; Department of Radiation Oncology, University of California San Francisco, San Francisco, CA, USA

**Keywords:** meningioma, multidisciplinary care, quality improvement, quality of life

## Abstract

**Background:**

Standard treatments for meningioma include surgery and radiotherapy, but the timing and technical details of these treatments are debated, particularly as advances in meningioma biology expand options for treatment. Patients with meningioma may experience adverse quality-of-life and neurocognitive sequelae following diagnosis and treatment, and survivorship resources for these patients are limited.

**Methods:**

We initiated a Multidisciplinary Meningioma Program (MMP) to advance the care of patients at our institution in September 2024. The MMP consists of synergistic clinical, scientific, and survivorship resources, including a weekly tumor board and multidisciplinary clinic, clinical trials and prospective registries for translational research, a meningioma-specific support group, and basic and translational research. In this article, we describe the implementation and initial patient-facing aspects of the MMP.

**Results:**

In the first 15 months of the MMP, 602 cases were discussed at the MMP tumor board, and 142 patients were seen in consultation in the MMP multidisciplinary clinic. Twelve percent of patients discussed in the MMP tumor board were offered clinical trial enrollment, and 84% were offered prospective registry enrollment. Participants in the MMP support group cited informational and emotional benefits. Following implementation of the MMP, the clinical volume of consultations for meningioma increased by 87.0%-533.3%, and the volume of treatments increased by 42.1%-115.2% across clinical departments.

**Conclusions:**

Through creation of a multidisciplinary program for meningioma treatment, research, and support services, we enabled consensus clinical recommendations, streamlined enrollment on clinical trials and prospective registries, improved access to support services, and increased patient volume.

Key Points

- A multidisciplinary program effectively integrates meningioma treatment, research, and support services
- Patients with meningioma can benefit from disease-specific clinical trials, prospective registries, and survivorship resources

Importance of the Study

Meningioma is the most common primary intracranial tumor. Given unique survivorship needs and the evolving landscape of molecular risk stratification, postoperative radiotherapy indications, and investigational systemic treatments, patients with meningioma may benefit from multidisciplinary management and coordination of care across healthcare providers and specialties. Here we report the University of California San Francisco (UCSF) Multidisciplinary Meningioma Program, which, to our knowledge, is the first meningioma-specific program to integrate a multidisciplinary clinic with a tumor board, clinical trials, prospective registries, translational research, and survivorship resources. Beyond highlighting a model that may improve care for patients with meningioma, we demonstrate tangible institutional benefits, including increased enrollment in clinical trials and increased patient volume. This program may serve as a model for other health systems to advance the care of patients with meningioma.

Meningioma accounts for 42.6% of newly diagnosed brain tumors in the United States.^1^ Maximal safe resection that minimizes neurologic morbidity is the gold standard of meningioma treatment.^2^ When surgical resection is contraindicated, incomplete, and/or reveals a high-grade tumor, fractionated radiotherapy can be an effective treatment option for meningioma and radiosurgery is often considered an appropriate alternative for small meningiomas.^3^^,^^4^ Radiotherapy modalities range from single-session stereotactic radiosurgery (SRS) to several weeks of conventionally fractionated ionizing radiation. In the absence of standardized treatment recommendations, the decision of when to operate or radiate, or whether to operate or radiate at all, is often left up to the discretion of individual healthcare providers. To date, systemic therapies are largely experimental but are increasingly used off-label for tumors that are refractory to standard interventions.^5^

Patients with meningioma can experience adverse health-related quality-of-life (HRQoL) outcomes, including physical symptoms and side effects, depression, anxiety, fatigue, difficulties in social and role functioning, and neurocognitive impairment, particularly in the domains of visual memory, executive function, and attention.^6-8^ These impairments can have a significant functional impact on patients and limit their ability to return to work.^9^ Investigations on the impact of standard-of-care meningioma treatment on patient-reported HRQoL and neurocognition are limited, though some studies suggest that baseline deficits may worsen after radiotherapy for meningioma.^10^ Because many patients with meningioma have excellent survival and may have persistent HRQoL impairments, their survivorship needs are distinct from those of many other patients with brain tumors. Integrative care planning, cognitive rehabilitation, and supportive care services devoted to patients with meningioma are needed.^11^

Given clinical equipoise in standard-of-care treatments for many meningiomas, the limited scope of preexisting research, the dearth of treatment options for meningiomas that are refractory to surgery and radiotherapy, and the evolving guidance of molecular analysis in treatment individualization, we hypothesized that patients with meningioma would benefit from robust multidisciplinary discussion, consistent incorporation of comprehensive next-generation sequencing, and expanded access to clinical trials, prospective registries, and survivorship resources.^11^^,^^12^ Here we report the practical considerations, clinical implementation, and preliminary outcomes of the patient-facing aspects of the University of California San Francisco (UCSF) Multidisciplinary Meningioma Program (MMP).

Prior to the creation and implementation of the MMP, patients with meningioma at our institution were seen in individual provider clinics without an integrated care plan. A preexisting adult central nervous system (CNS) tumor board allowed for some discussion of the management of complex clinical cases, but evolving meningioma molecular classification systems and patient volume did not allow for comprehensive discussion. Thus, clinical recommendations were often fragmented and lacked broad multidisciplinary consensus for the majority of patients with meningioma, and preexisting survivorship programming in the UCSF Division of Neuro-Oncology focused on patients with glioma. The MMP radically changed the logistics and resources for treatment, research, and support services for patients with meningioma at UCSF and may serve as a model for similar programs at other institutions to advance the care of patients with the most common primary intracranial tumor.

## Methods

### Multidisciplinary Meningioma Tumor Board

In a pilot effort to streamline multidisciplinary meningioma care in September 2024, we convened a Multidisciplinary Meningioma Tumor Board (MMTB) to provide a forum for the discussion of complex clinical cases. The MMTB consisted of a weekly one-hour meeting of meningioma healthcare providers, including physicians, nurse practitioners, nurse navigators, and clinical research coordinators (CRCs) from the Departments of Neurological Surgery, Radiation Oncology, Radiology, Pathology, and Ophthalmology, as well as the Divisions of Neuro-Oncology and Neuro-Psychology. A member of each referred patient’s care team was required to attend the MMTB meeting to provide context regarding the patient’s clinical status, goals of care, and past medical history. The principal investigators of any open clinical trials for patients with meningioma also regularly attended the MMTB to provide insight into eligibility and accelerate patient referrals. Each week, a neuropathologist and neuroradiologist reviewed referred cases before the MMTB meeting and presented diagnostic data alongside the clinical information and questions presented by referring providers. A consensus recommendation incorporating input from providers in Neurological Surgery, Radiation Oncology, Neuro-Oncology, Radiology, and Pathology was made for all cases presented before the MMTB, and the recommendation was conveyed to the patient’s care team and documented in the electronic medical record.

Cases were referred to the MMTB through internal provider request, external referral, or through pathologic and genetic screening of meningioma tissue samples. Every meningioma that is surgically resected at UCSF undergoes next-generation DNA sequencing via the UCSF500 assay to allow for accurate molecular classification and identification of targetable mutations.^13^ The MMTB neuropathologist reported sequencing results ahead of each weekly meeting, and the program CRC prescreened sequencing results for cases in need of group discussion (Table 1). World Health Organization (WHO) CNS grade 1 meningiomas with favorable genetics after gross total resection were omitted from discussion, given broad consensus on standard-of-care postoperative surveillance for such cases. Whether discussed at the MMTB or not, all postoperative patients were referred to Neuro-Oncology for discussion of tumor genomics.

**Table 1.** Grading and genetics of consecutive meningiomas screened for the weekly multidisciplinary meningioma tumor board.

	All	Discussed	Triaged	Pathology consultation
Meningiomas	576	225	186	164
WHO grade				
1	277 (48.1%)	94 (41.8%)	124 (66.7%)	57 (34.8%)
2	199 (34.5%)	97 (43.1%)	42 (22.6%)	60 (36.6%)
3	47 (8.2%)	23 (10.2%)	13 (7.0%)	10 (6.1%)
Undefined	53 (9.2%)	11 (4.9%)	7 (3.8%)	37 (22.6%)
Copy number alterations^a^				
Chr22q deletion	309 (53.6%)	101 (44.9%)	96 (51.6%)	112 (68.3%)
Chr1p deletion	207 (35.6%)	92 (40.9%)	46 (24.7%)	69 (42.1%)
Chr14q deletion	122 (21.2%)	49 (21.8%)	30 (16.1%)	43 (26.2%)
Chr6q deletion	91 (15.8%)	42 (18.7%)	19 (10.2%)	30 (18.3%)
Chr18q deletion	77 (13.4%)	30 (13.3%)	16 (8.6%)	31 (18.9%)
Chr1q duplication	57 (9.9%)	23 (10.2%)	17 (9.1%)	17 (10.4%)
Chr10q deletion	58 (10.1%)	22 (9.8%)	12 (6.5%)	24 (14.6%)
Chr18p deletion	58 (10.1%)	21 (9.3%)	12 (6.5%)	25 (15.2%)
Chr10p deletion	51 (8.9%)	19 (8.4%)	10 (5.4%)	24 (14.6%)
Chr20q duplication	48 (8.3%)	10 (4.4%)	14 (7.5%)	24 (14.6%)
Chr17q duplication	46 (8.0%)	13 (5.8%)	12 (6.5%)	21 (12.8%)
Chr6p deletion	43 (7.5%)	10 (4.4%)	14 (7.5%)	19 (11.6%)
Chr20p duplication	42 (7.3%)	11 (4.9%)	10 (5.4%)	21 (12.8%)
Chr4p deletion	38 (6.6%)	9 (4.0%)	12 (6.5%)	17 (10.4%)
Chr7p deletion	36 (6.3%)	13 (5.8%)	8 (4.3%)	15 (9.1%)
Chr2p deletion	35 (6.1%)	12 (5.3%)	7 (3.8%)	16 (9.8%)
Chr3p deletion	35 (6.1%)	11 (4.9%)	7 (3.8%)	17 (10.4%)
Chr5p duplication	35 (6.1%)	8 (3.6%)	9 (4.8%)	18 (11.0%)
Chr5q duplication	36 (6.3%)	9 (4.0%)	9 (4.8%)	18 (11.0%)
Chr4q deletion	34 (5.9%)	10 (4.4%)	9 (4.8%)	15 (9.1%)
Chr19p deletion	33 (5.7%)	18 (8.0%)	9 (4.8%)	6 (3.7%)
Chr8p deletion	32 (5.6%)	14 (6.2%)	4 (2.2%)	14 (8.5%)
Chr9p deletion	32 (5.6%)	15 (6.7%)	8 (4.3%)	9 (5.5%)
Chr11p deletion	31 (5.4%)	11 (4.9%)	7 (3.8%)	13 (7.9%)
Chr12q duplication	30 (5.4%)	5 (2.2%)	8 (4.3%)	17 (10.4%)
Somatic short variants^b^				
*NF2*	253 (43.9%)	85 (37.8%)	83 (44.6%)	85 (51.8%)
*TRAF7*	106 (18.4%)	27 (12.0%)	48 (25.8%)	31 (18.9%)
*KLF4*	43 (7.5%)	14 (6.2%)	19 (10.2%)	10 (6.1%)
*AKT1*	29 (5.0%)	6 (2.7%)	11 (5.9%)	12 (7.3%)
*SMARCB1*	20 (3.5%)	6 (2.7%)	8 (4.3%)	6 (3.7%)
*TERTp*	17 (3.0%)	7 (3.1%)	4 (2.2%)	6 (3.7%)
*CDKN2A*	17 (3.0%)	8 (3.6%)	5 (2.7%)	4 (2.4%)
*PIK3CA*	16 (2.8%)	5 (2.2%)	6 (3.2%)	5 (3.0%)
*CDKN2B*	14 (2.4%)	8 (3.6%)	2 (1.1%)	4 (2.4%)
*ARID2*	13 (2.3%)	4 (1.8%)	7 (3.8%)	2 (1.2%)
*ARID1A*	11 (1.9%)	2 (0.9%)	4 (2.2%)	5 (3.0%)
*SMO*	10 (1.7%)	2 (0.9%)	5 (2.7%)	3 (1.8%)
*POLR2A*	10 (1.7%)	2 (0.9%)	6 (3.2%)	2 (1.2%)
*TET2*	8 (1.4%)	2 (0.9%)	5 (2.7%)	1 (0.6%)
*CREBBP*	8 (1.4%)	4 (1.8%)	2 (1.1%)	2 (1.2%)
*CHEK2*	8 (1.4%)	2 (0.9%)	3 (1.6%)	3 (1.8%)
*TP53*	7 (1.2%)	4 (1.8%)	0 (0.0%)	3 (1.8%)
*KDM6A*	6 (1.0%)	4 (1.8%)	1 (0.5%)	1 (0.6%)
*KMT2D*	6 (1.0%)	4 (1.8%)	1 (0.5%)	1 (0.6%)

a Copy number alterations reported if observed in ≥5% of resected meningiomas.

b Somatic short variants reported if observed in ≥1% of resected meningiomas.

### Multidisciplinary Meningioma Clinic

Alongside the weekly MMTB, we convened a weekly Multidisciplinary Meningioma Clinic (MDMC) that was staffed by a neurosurgeon, a radiation oncologist, and a neuro-oncologist. These providers collaboratively consulted on 3-5 cases each week, which were selected for multidisciplinary consultation based on complexity and/or the intrinsic need for multiple healthcare specialists to facilitate treatment recommendations, such as for SRS alone or SRS and immunotherapy on a clinical trial, which is described in greater detail below. The MDMC met immediately following MMTB on the same day to ensure efficient delivery of individualized consensus recommendations. The MDMC conducted patient consults in clinic rooms in the Division of Neuro-Oncology or over telemedicine. Whenever possible, patients were offered both in-person and virtual appointments to improve accessibility. No new physicians were hired for the MDMC. Rather, the percent effort required to orchestrate and carry out the MDMC was absorbed by existing physicians who were allocated time to do so by their respective departments or divisions, which amounted to approximately one half-day per week.

### Administrative Support Staff

Each meeting of the MMTB and MDMC was attended by a nurse navigator and a CRC who were specifically hired to support the MMP. These were the only new positions created and funded to support the MMP. The CRC compiled the weekly MMTB list for discussion from individual provider requests and postoperative cases. The nurse navigator supported the MMTB and MDMC by assembling medical records from other institutions, scheduling patients in the MDMC, documenting MMTB consensus recommendations in the electronic medical record, and coordinating referrals to other healthcare providers after MMTB discussion. If the MMTB recommended MDMC referral, the nurse navigator followed up with the patient to schedule an appointment. The nurse navigator also called patients in the morning of MDMC appointments to confirm patient attendance ahead of consultation.

Following the weekly MMTB meeting, the MMP CRC consented all eligible patients for prospective registries, including a HRQoL study and a biomarker development study, which are described in further detail below. Every week, the CRC monitored the MDMC and individual provider clinic schedules to consent patients after existing appointments whenever possible, thereby minimizing patient inconvenience and attrition. If patients did not have upcoming clinic appointments, they were messaged over MyChart to schedule a phone conversation to discuss prospective registry enrollment. When meeting with the CRC, if patients expressed emotional strain or a desire for additional support, they were directed to preexisting neuro-oncology survivorship resources that are available to all brain tumor patients at UCSF, such as an integrative health webinar series, peer-to-peer phone conversations, exercise and wellness classes, and a neurocognitive rehabilitation clinic. The MMP CRC also offered all patients enrollment in a meningioma-specific support group, which was initiated to provide specialized supportive care as part of the MMP and is described in greater detail below. The support group was further promoted through informational posters in every neuro-oncology, neurosurgery, and radiation oncology clinic room.

### Meningioma Clinical Trials

In the first 15 months of the MMP, patients referred to clinical trials were offered either the SRS-AIM study of adjuvant hypofractionated radiosurgery for intermediate-risk meningioma (NCT06557512) or a study of combined SRS and pembrolizumab for recurrent, high-grade meningiomas (NCT04659811), which were open to enrollment during this period. The former study was open to patients with gross total surgical resection of a recurrent WHO grade 1 meningioma or a newly diagnosed WHO grade 2 meningioma. The latter study was open to patients with recurrent WHO grade 2 or 3 meningioma that was amenable to radiosurgery. Therefore, these clinical trials were open to completely non-overlapping patient populations.

### Meningioma Prospective Registries

All eligible patients discussed in the MMTB were offered enrollment in 2 prospective registries: one for prospective validation of a targeted gene expression biomarker and the development of new biomarkers and the other for longitudinal HRQoL assessment. The Biomarker Evaluation for All Meningioma (BEAM) study aims to use prospective tumor samples to validate a 34-gene expression biomarker that was previously developed using a retrospective discovery cohort of 173 meningiomas from UCSF and validated in more than 2000 retrospective meningioma samples from 13 medical centers across 3 continents.^14^^,^^15^ The gene expression biomarker has previously been shown to improve outcome discrimination compared to standard-of-care histological and molecular assessments and to refine postoperative management for 29.8% of patients, including identifying 19.8% of clinical low-risk meningiomas with unfavorable local freedom from recurrence.^14^^,^^15^ The Living With Meningioma (LWM) HRQoL study investigates the impacts of meningioma diagnosis and treatment on patient-reported outcomes through a variety of validated quality-of-life, symptom burden, and financial toxicity questionnaires, including EORTC QLQ-C30, QLQ-BN20, FACIT-COST, and the Fatigue Symptom Inventory, as well as a series of meningioma-specific surveys on treatment and health history, including a questionnaire on anxiety adapted from the Memorial Anxiety Scale for Prostate Cancer.^16^^,^^17^

### Meningioma Support Group

To address the distinct HRQoL needs of patients with meningioma, a monthly virtual meningioma-specific support group was initiated in June 2025. The support group was co-led by the MMP CRC and nurse navigator to foster patient familiarity and comfort through continuity of support staff. Meeting structure was designed based on guidance from the literature, with consultation from the facilitators of our institution’s preexisting general brain tumor support group and a local prostate cancer support group, both of which have met for over two decades.^18^ The group met for a duration of 1.5 hours per session and was open to any UCSF patient with a meningioma diagnosis. Each session opened with a brief, facilitator-led presentation on meeting agreements, including the confidential nature of the discussion and prohibition on offering medical advice. Following this introduction, participants were invited to respond to open-ended prompts, including “What is your meningioma story?”, “What is your biggest concern right now?”, and “What are some questions you have for the group?”. Each participant was given four minutes to introduce themselves and share their response to these prompts. Patients were also permitted to opt-out of sharing if preferred. Once every interested participant had shared, the facilitators asked participants to recall themes and questions that came to mind during the share session. Participants were then invited to discuss these meeting topics in an open forum with no imposed share time for the remainder of the meeting. A percentage of CRC and nurse navigator effort was directed toward the meningioma support group. Otherwise, there were no costs associated with organizing or hosting this group.

### Statistical Methods

Descriptive statistics, including mean, median, and rate, and measures of variability, including range and 95% confidence interval, were compiled for patient volume metrics in the MMTB, the MDMC, clinical trials, and prospective registries such as the LWM HRQoL study. Linear regression for patient volume per year by diagnosis in the Division of Neuro-Oncology was conducted using the lm() package in R v4.2.2. Homogeneity of regression slopes was compared using the ANCOVA test in the lm() package in R v4.2.2. A threshold of *P* < .05 was considered statistically significant.

## Results

### Multidisciplinary Meningioma Tumor Board and Clinic

**Figure 1.**
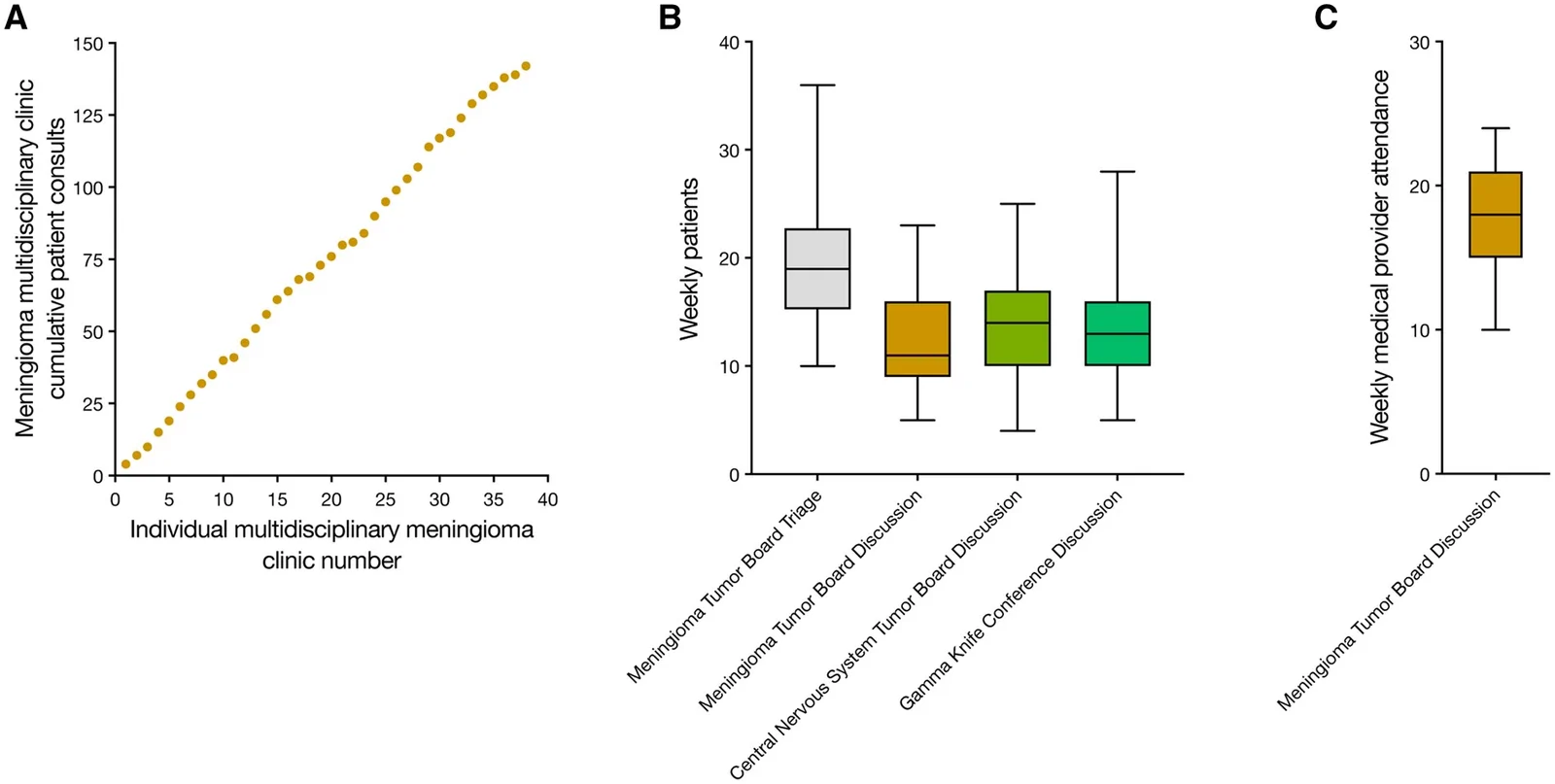
Patient and provider discussions, clinic visits, and attendance at the multidisciplinary meningioma tumor board and clinic. (A) Cumulative patient consultations in the UCSF multidisciplinary meningioma clinic from September 2024 to November 2025. (B) Weekly patients triaged or discussed at the meningioma, central nervous system, or Gamma Knife tumor boards at UCSF from September 2024 to November 2025. (C) Weekly healthcare provider attendance at the UCSF multidisciplinary meningioma tumor board from September 2024 to November 2025. Lines represent the mean, boxes represent second and third quartiles, and error bars represent the range.

Over a 15-month period from September 2024 to November 2025, the MMTB discussed a median of 13 patients per week (range 11-24). In total, 602 cases representing 399 unique patients were discussed from September 2024 to November 2025 (Table 2), and 142 patients were seen in consultation in the MDMC during this period (Figure 1A). Two preexisting adult CNS tumor boards at UCSF, one for all adult brain tumors (median 14 patients per week, range 4-25) and the other specifically for consideration of SRS (median 13 patients per week, range 5-28), discussed comparable numbers of patients during this period, supporting the need for a distinct, meningioma-specific tumor board meeting (Figure 1B). An additional 167 cases were triaged out of MMTB discussion based on benign clinical, histologic, and molecular features or if repeat discussion would not change management. One hundred fifty-one pathology and molecular consultations from external institutions were also triaged out of discussion due to the lack of clinical context from incomplete medical records. Of the patients discussed in the MMTB, 66% were female, with a median age of 63 (range 14-89). The WHO grades of meningiomas discussed were 36% grade 1, 33% grade 2, 8% grade 3, and 23% ungraded (e.g., imaging-defined or intermediate-grade).

**Table 2.** Demographics and clinical history of patients discussed in the weekly multidisciplinary meningioma tumor board.

	Patients
Discussions per patient	
1	243 (60.9%)
2	108 (27.1%)
3	37 (0.93%)
4+	11 (0.03%)
Sex	
Female	265 (66.4%)
Male	134 (33.6%)
Median age (range)	63 (14-89)
First discussion presentation	
Newly diagnosed	258 (64.7%)
Recurrent	141 (35.3%)
Multiple meningioma	106 (26.6%)
Prior treatment	
Surgery	285 (71.4%)
Radiotherapy	110 (27.6%)

A median of 20 healthcare providers attended the MMTB each week (range 11-24) (Figure 1C), which often included multiple physicians from each discipline of Neurological Surgery, Radiation Oncology, and Neuro-Oncology, who contributed to consensus clinical recommendations. MMTB recommendations consisted of radiosurgery (36%), observation (30%), fractionated radiotherapy (21%), surgery (19%), systemic therapy (16% of all patients, 36% of patients with WHO grade 2 or 3 meningioma), and additional work-up with SSTR2-directed PET imaging (9%). Thirty-three percent of patients discussed were referred to the MDMC, and 71% of patients referred to the MDMC were seen in consultation on the same day as MMTB discussion. Twelve percent of patients discussed were offered clinical trial enrollment, and 84% were offered prospective registry enrollment. Of patients who discussed prospective registry enrollment with the program CRC, 77% consented to one or both available studies.

### Meningioma Clinical Trials

From initiation in March 2021 to completion in February 2026, 34 patients consented to the SRS and immunotherapy study (NCT04659811). From March 2021 to September 2024, a period of roughly 3.5 years prior to the creation and implementation of the MMP, 20 patients were enrolled in this study. From the creation of the MMP in September 2024 to February 2026, a period of 18 months, 14 additional patients were enrolled. Hence, the rate of accrual on this trial increased from 5.7 patients per year to 9.3 patients per year after the creation and implementation of the MMP. Of the 14 patients enrolled in this study after the creation and implementation of the MMP, 13 patients (92.8%) were recommended for study enrollment during MMTB discussion. The SRS-AIM study of hypofractionated SRS for intermediate-risk meningiomas (NCT06557512) has enrolled 6 patients since initiation in February 2025, all of whom were recommended for study enrollment during MMTB discussion.

One patient enrolled in the SRS and immunotherapy study experienced a rapid, dramatic reduction in tumor volume, likely modulated by a rare meningioma DNA mismatch repair mutation that was identified on next-generation UCSF500 DNA sequencing.^19^ This case illustrates the importance of clinical trials and molecular profiling for patients with limited treatment options in a comprehensive care program for meningioma.

### Meningioma Prospective Registries

Two hundred seventy-one patients with tumor samples available at our institution were consented for the BEAM study from September 2024 to November 2025 through the MMP. Through prospective biomarker validation, the BEAM study is laying the groundwork for the incorporation of molecular risk stratification into MMTB discussions to enhance individualization of treatment recommendations. From September 2024 to February 2026, 151 patients consented to and participated in the LWM HRQoL study. Among the 151 initial participants, composite physical (mean 85.34, 95% confidence interval [CI], 81.74-88.86), cognitive (mean 77.31, 95% CI, 73.61-81.01), and emotional (mean 72.19, 95% CI, 68.42-75.96) functioning appeared lower than that of normative populations.^20^ Among employed participants (*n* = 113), 41.6% (*n* = 47) reduced work hours after diagnosis and treatment, and, of those, 60.9% (*n* = 28) had not returned to previous work levels, corroborating difficulties with employment and return to work that have been reported in the literature for patients with meningioma.^9^ In further support of this finding, 30.4% of participants who completed a financial distress questionnaire (FACIT-COST) (*n* = 45/148) reported financial toxicity (FACIT-COST <26). Though these data are preliminary and unstratified from an ongoing study and therefore may have limited clinical significance, they provide insight into areas of HRQoL and survivorship interventions that may benefit patients with meningioma.

### Meningioma Support Group

In response to the preliminary data from the LWM HRQoL study, we initiated a meningioma-specific support group. Patients reported hearing about the support group from providers, social media, the internet, nonprofit organizations, and supportive care resources of healthcare institutions. This outreach has garnered considerable interest, with 95 patients subscribed to the support group mailing list as of April 2026. Sixty-one unique patients attended the group’s first eleven meetings, with a median of 13 patients (range 9-18) attending each month. Thirty-five individuals attended more than one meeting of the support group. When surveyed, 95% of respondents (*n* = 18/19) rated the support group as good or excellent, and 84% (*n* = 16/19) attested that they were likely to attend the group again. When asked to select from a list of the support group’s possible benefits, 58% (*n* = 11/19) of patients selected “useful information,” 53% (*n* = 10/19) selected “a space to share,” 37% (*n* = 7/19) selected “a sense of community,” and 32% (*n* = 6/19) selected “emotional support.”

### Consultation and Treatment Volume

**Figure 2.**
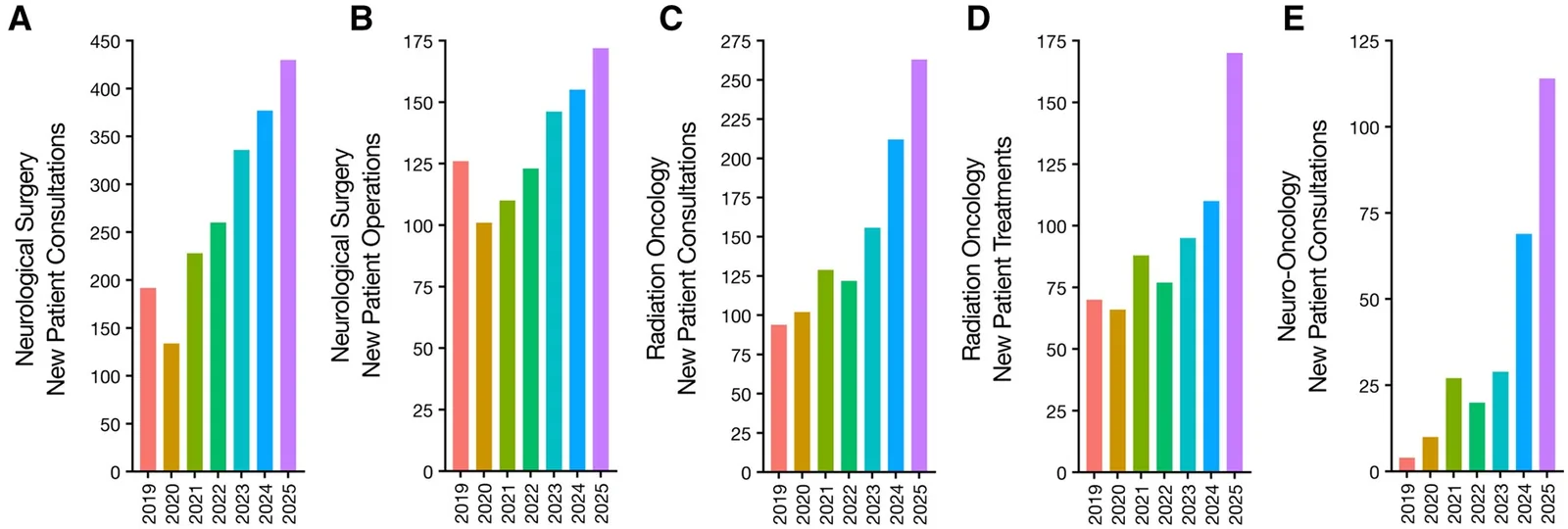
Patient volume before and after creation and implementation of the multidisciplinary meningioma program in September 2024. Annual new patient consults or treatments for meningioma in (A and B) the UCSF Department of Neurological Surgery, (C and D) Department of Radiation Oncology, and (E) Division of Neuro-Oncology.

Analyses of the annual number of new patients with meningioma at UCSF demonstrated significant increases in consultations and treatments since the creation and implementation of the MMP in September 2024. In the Department of Neurological Surgery, the number of new consultations for meningioma increased by 87.0% (*n* = 430) and the number of new operations for meningioma increased by 42.1% (*n* = 172) in 2025 compared to the average number of annual consultations (230, range 134-336) and operations (121, range 101-146) in the 5 years preceding the MMP (2019-2023) (Figure 2A and B). In the Department of Radiation Oncology, the number of new consultations for meningioma increased by 117.4% (*n* = 263) and the number of new treatments for meningioma increased by 115.2% (*n* = 170) in 2025 compared to the previous average number of annual consultations (121, range 92-156) and treatments (79, range 66-95) from 2019 to 2023 (Figure 2C and D). In the Division of Neuro-Oncology, the number of new consultations for meningioma increased by 533.3% (*n* = 114) in 2025 compared to the previous average number of annual new consultations from 2019 to 2023 (18, range 4-29) (Figure 2E).

Data regarding new consultation volume for IDH-mutant glioma and IDH-wildtype glioblastoma were available from the Division of Neuro-Oncology from 2023-2025. The volume of new patient consults in Neuro-Oncology for IDH-mutant gliomas increased from 102 in 2023 to 137 in 2024 and 197 in 2025, representing an average linear increase of 46.6% per year. The volume of new patient consults for IDH-wildtype glioblastoma increased from 199 in 2023 to 269 in 2024 and 369 in 2025, representing an average linear increase of 42.7% per year. Pooling these data gives an estimate of an average annual increase of 44.6%. In contrast, the volume of new patient consults for meningioma increased from 29 in 2024 to 69 in 2024 and 114 in 2025, representing an average linear increase of 146.6% per year after the introduction of the MMP. The yearly increase in meningioma consults was higher than the yearly increase in pooled glioma consults (*P* = .0055 by ANCOVA test of homogeneity of regression slopes). Using the increase in glioma patient volume as a benchmark representing post-pandemic recovery and baseline institutional growth, meningioma patient volume growth outpaced these factors, and a substantial proportion of the excess growth is likely attributable to the introduction of the MMP.

## Discussion

Meningiomas are the most common primary intracranial tumor in adults, and multidisciplinary programmatic efforts can advance care, catalyze research, and develop support services for this patient population. Here we report a new multidisciplinary meningioma program that, to our knowledge, is the first meningioma-specific program to integrate a multidisciplinary clinic with a specialized tumor board, clinical trials, prospective registries, translational research, and survivorship resources. The centralization of these efforts at our institution allowed for clinical consensus on recommendations, consistent application of molecular risk stratification, facile and accelerated clinical trial enrollment, and a greater understanding of patient-reported outcomes, all of which were previously not routinely available for patients with meningioma. In the prospective LWM HRQoL study, we identified physical, cognitive, and emotional functioning deficits, work reduction, and financial toxicity among study participants. These initial data accord with previously reported findings, including those from the “Quality of life outcomes in incidental and operated meningiomas” (QUALMS) study, a large single-center cross-sectional HRQoL study of 243 patients who underwent surgery or active surveillance for meningioma.^21^ With almost a decade of follow-up, this study found significant impairments in multiple domains among patients with meningiomas compared to the normative population, including in physical, cognitive, and role functioning, and found exacerbation of symptoms such as fatigue and pain.^21^ These data highlight the impact of meningioma diagnosis and treatment on patients and underscore the need for meningioma-specific survivorship resources. Acknowledging this need, we created a support group within the MMP, which was reviewed favorably and perceived as beneficial by participants.

Taken together, these integrated programmatic efforts culminated in increased patient volume across multidisciplinary health care specialties. For example, the growth in meningioma patient volume in the Division of Neuro-Oncology increased more than 500% and significantly exceeded that of IDH-mutant glioma and IDH-wildtype glioblastoma over the same period. Although this analysis was limited to a 3-year period from a single division due to constraints of accessible data, this finding suggests that the growth in meningioma patient volume was not merely the result of other factors, like deferred healthcare during the COVID-19 pandemic and/or baseline institutional growth. Since no other significant institutional changes in meningioma care were implemented during this interval, we attribute the excess growth primarily to the introduction of the MMP. External factors, such as referral patterns, could have contributed to non-uniform increases in patient volume, and changes in provider and administrator staffing and percent effort should also be considered. Nevertheless, these initial data that suggest significant growth in patient volume are encouraging.

From September to December 2024, the MMTB and MDMC met twice a month before it was determined that clinical volume was too great to sustain a bimonthly meeting in a one-hour format. Therefore, meeting frequency was increased to weekly in January 2025. The MMTB list was initially compiled by a physician until the caseload demonstrated that a CRC was necessary for these administrative tasks. Similarly, scheduling for the MDMC was originally undertaken by preexisting staff in the Division of Neuro-Oncology until the quantity of patients referred to the MDMC exceeded staff capacity and a dedicated nurse navigator was recruited. Difficulties in obtaining insurance authorization for simultaneous consultation with three healthcare providers, as well as cumbersome patient transitions between individual provider clinics and the MDMC, also underscored the necessity of a full-time nurse navigator for the MMP. Through these challenges, we came to understand the importance of dedicated, full-time support staff for the successful implementation of an MMP.

To date, the CRC and nurse navigator have been the only new positions created and filled to sustain the MMP. Physician and other clinic administrator duties were absorbed by existing staff as a percentage of their existing effort, and their compensation was derived from usual means. The costs of creating similar multidisciplinary programs at other institutions may vary, primarily due to differences in salary requirements for new positions that vary between institutions and by location-specific cost of living. In the framework we have established, however, as few as 2 additional personnel may be required, indicating potentially broad feasibility. Depending on factors such as reimbursement and payor mix, the increase in patient volume generated by a multidisciplinary program may justify the personnel investment in some health systems. Aside from CRC and nurse navigator time and effort, other components of our MMP have no associated costs. Though the unique constraints of other healthcare models may change the economics and feasibility of implementing similar programs, the patient-facing benefits of streamlined, individualized care alongside enhanced access to clinical trials and survivorship resources are expected to be more universal.

The present analysis has several limitations that warrant consideration. Quantitative metrics describing clinical consensus and decision-making before the introduction of the MMP were not available, which limits our ability to directly measure the impact of the MMP on these aspects of patient care. Instead, the impacts of the MMP on clinical consensus and its benefit to patients were described qualitatively here. Additionally, the recency of our program does not currently allow for evaluation of the impact of the MMP on disease-specific clinical outcomes, like progression-free survival or overall survival, or patient-reported HRQoL outcomes. As longitudinal data mature from patients managed by the MMTB and MDMC, as well as from those involved in the meningioma-specific support group and LWM HRQoL study, we can more rigorously analyze the effect this model has on clinical and patient-reported outcomes.

Beyond continued longitudinal observation and evaluation, we plan to expand this program through additional prospective registries, therapeutic clinical trials, and pilot programs in the coming years. These new initiatives include (1) a longitudinal pre- and post-operative neurocognitive evaluation protocol for patients with meningioma; (2) a quarterly patient-facing webinar series; (3) a study examining biological and social drivers of racial disparities in meningioma prevalence and severity (NCT07418775), including the impact of progestin-based female contraception on meningioma growth^22^; and (4) in-person support group events. During the development of these projects, we have consulted with the UCSF Cancer Center’s Community Advisory Board to obtain feedback on the accessibility and relevance of our work to underserved communities. Through collaboration with local leaders and organizations on study design and supportive care offerings, we hope to further integrate our program’s mission with the needs of the broader community of patients who are living with meningioma.
